# The road toward a physiological control of artificial respiration: the role of bio-inspired neuronal networks

**DOI:** 10.3389/fnins.2025.1638547

**Published:** 2025-08-28

**Authors:** Federica Perricone, Lorenzo Tartarini, Lorenzo De Toni, Luigi Rovati, Jonathan Mapelli, Daniela Gandolfi

**Affiliations:** ^1^Department of Biomedical, Metabolic and Neural Sciences, University of Modena and Reggio Emilia, Modena, Italy; ^2^Department of Engineering “Enzo Ferrari”, University of Modena and Reggio Emilia, Modena, Italy; ^3^Center for Neuroscience and Neurotechnology, University of Modena and Reggio Emilia, Modena, Italy

**Keywords:** artificial breathing, computational neuroscience, respiratory CPG, neuronal simulation, real-time closed loop controls

## Abstract

The transition from mechanical to physiological ventilation is a delicate step during the recovery from ECMO, in particular following severe respiratory failures. Since there is controversy on the optimal degree of mechanical ventilation support, the maintenance of physiological ventilation can be crucial to determine the balance between lung rest and lung recovery. We believe that the development of closed-loop control systems for mechanical ventilation, designed to maintain or restore physiological respiratory activity in patients supported by extracorporeal membrane oxygenation (ECMO) could contribute to achieve this goal. In our vision, the core of such a system could be a biologically inspired computational model of the respiratory neural control center, capable of simulating the respiratory rhythm required to efficiently eliminate CO₂ from the body. The outputs of the modeled respiratory rhythm (e.g., rate and pattern) would represent the patient’s needs that should be ideally maintained to ensure proper CO₂ clearance. The use of a simulated respiratory rhythm to dynamically control a mechanical ventilator integrated with ECMO would ensure that ventilatory support is adjusted in real time to meet the physiological demands indicated by inputs delivered by external sensors. One of the key advantages of this system would be its use during weaning from ECMO. By simulating a target respiratory rhythm and gradually transferring the workload from ECMO to mechanical ventilation, the system could allow for a smoother and safer transition to spontaneous or assisted breathing.

## Introduction

When spontaneous breathing fails or is compromised, one of the main clinical challenges is to adopt a strategy that allows the replication of breathing to reproduce the physiological behavior. Progress toward models that account for the neuronal generation of the respiratory rhythm and can receive and provide inputs in real time may help establish a direct relationship between the patient’s conditions and the breathing activity necessary to meet their physiological demands.

**Figure 1:**
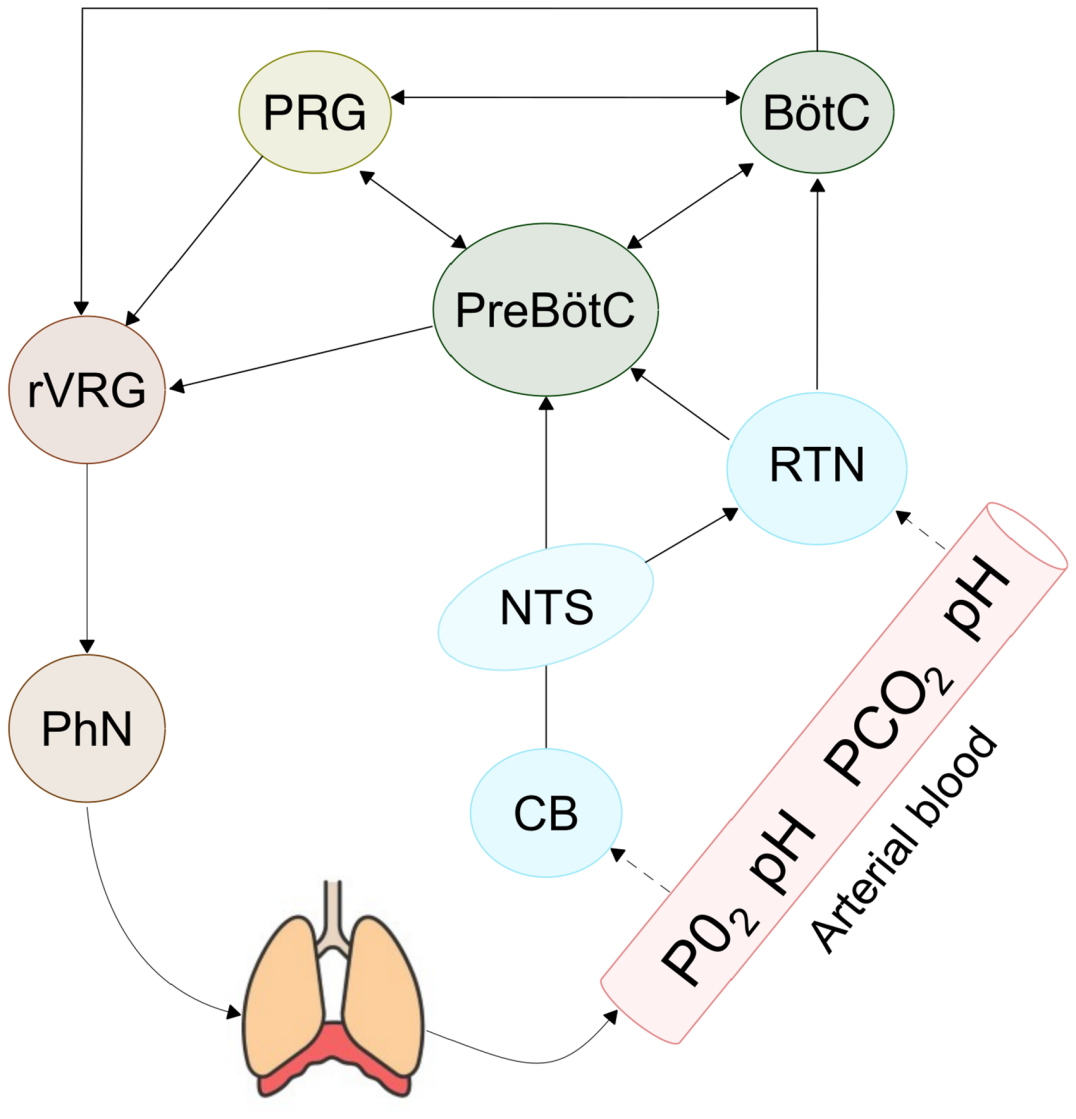
Schematic representation of the core nuclei of the respiratory circuit. The RTN and carotid bodies detect changes in pH and PaCO_2_ and PaO_2_, respectively, and relay these modulatory signals to the PreBötC and the BötC. Together with the PRG, PreBötC, BötC form the CPG, which orchestrates inspiration, active expiration, and the transitions between respiratory phases. The output from the CPG nuclei is conveyed to the rostral ventral respiratory group (rVRG), which transmits the rhythmic respiratory drive to the phrenic nerve (PhN), thereby initiating and sustaining the breathing cycle.

The respiratory rhythm is an oscillatory event which arises from a specialized neuronal circuit called central pattern generator (CPG). CPGs are structures present in multiple nervous centers and distributed in the human body which produce rhythmic activity, such as walking, swimming, and chewing, without requiring afferent input. Specifically, the respiratory CPG (rCPG) is a neuronal circuit whose emergent activity drives the respiratory muscles responsible for breathing. The rCPG is primarily localized in the pons and medulla of the brainstem, coordinating the respiratory cycle composed by inspiration, post inspiration, and expiration. These phases have traditionally been considered as a single integrated rhythm, whereas recent studies associate them with three distinct coupled oscillators, with inspiration being the only fundamental phase of the cycle (Del Negro et al., 2018). The generation of inspiratory rhythm can be traced anatomically to the preBötzinger Complex (preBötC), a cluster of neurons in the ventrolateral medulla (Schwarzacher et al., 2011). This heterogeneous population comprises excitatory glutamatergic neurons and inhibitory GABAergic and glycinergic neurons whilst experimental studies have shown that only excitatory neurons are necessary and sufficient to generate respiratory rhythm (Janczewski et al., 2013). Despite the preBötC is widely recognized as the rhythmogenic kernel of respiration, the precise mechanisms underlying autorhythmic activity remain under debate. A central question is whether the respiratory rhythm is primarily generated by intrinsic cellular properties, as proposed by the pacemaker theory, or whether it emerges from network dynamics, as suggested by the Burstlet theory (Smith et al., 1991; Kam et al., 2013). According to the latter, spontaneous activity in a subset of excitatory neurons gives rise to weakly synchronized events, called burstlets, which, once sufficiently synchronized, evolve into full population bursts capable of initiating inspiration (Kallurkar et al., 2020; Ashhad and Feldman, 2020). In contrast to pacemaker theory, burstlet theory does not require neurons that autonomously generate bursts of action potentials; instead, it posits that rhythmogenesis emerges as a property of the preBötC network, driven by excitatory feedforward synaptic interactions. Numerous experimental and computational studies have attempted to clarify these hypotheses. In particular, computational models based on the Hodgkin Huxley formalism have been used to explore whether rhythmogenesis can be driven by intracellular Ca^2+^ concentration (Rubin et al., 2009), Sodium persistent voltage dependent current (Phillips and Baertsch, 2024) or extracellular potassium accumulation (Phillips and Rubin, 2022). A recent study (Phillips and Baertsch, 2024) proposed a unifying model, demonstrating that rhythmogenesis arises from the interdependence between a persistent sodium current and recurrent excitation. While these biophysically detailed models provide valuable insights for the study of rhythmogenesis, their high computational cost poses a significant limitation when scaling up to larger networks or when incorporating multiple interacting populations into full-scale circuit models. In fact, the architecture that orchestrates respiratory rhythm is remarkably complex (Krohn et al., 2023), involving neurons from multiple brain areas (see Figure 1). Among these, the ventral respiratory group (VRG) contributes to inspiration through the preBötC and to expiration via the Bötzinger complex (BötC), a cluster of inhibitory neurons that regulates active expiration during exercise or speech. The pontine respiratory group (PRG) regulates the transitions between inspiration and expiration, contributing to the smooth progression of the respiratory cycle and it is also involved in coordinating complex behaviors, such as vocalization, swallowing, and the modulation of respiratory patterns during emotional states.

The respiratory rhythm is further modulated by several structures that send input to the CPG to dynamically adjust the respiratory rate and maintain homeostasis. One key structure is the retrotrapezoid nucleus (RTN), also known as the lateral parafacial due to its anatomical proximity to the facial nucleus. The RTN plays a pivotal role in central chemoreception, as it consists of neurons that detect changes in arterial pH and CO_2_ levels. In response to hypercapnia in fact, RTN neurons increase their firing rate and send excitatory inputs to the preBotC and other nuclei within the VRG, to promote the increase of ventilation required to expel excess CO_2_ and to restore physiological pH (Guyenet et al., 2019). Another key area that regulates metabolic homeostasis is the nucleus of the solitary tract (NTS), located in the dorsal respiratory group (DRG). The NTS integrates afferent input from peripheral chemoreceptors in the carotid bodies, which detect fluctuations in O_2_, CO_2_ and pH levels, and contributes to respiratory regulation during hypoxia and hypercapnia. The NTS sends widespread projections to both brainstem and forebrain regions involved in respiratory and behavioral regulation, including key respiratory centers such as the preBötC and BötC, the RTN, and other components of the VRG.

Computational models are increasingly becoming fundamental tools to endeavor the understanding of the dynamical operations performed by the respiratory control system and three types of models have been developed to attempt reproducing the activities involved in the generation and modulation of the respiratory pattern: biologically realistic (Phillips and Baertsch, 2024; Mapelli et al., 2021), integrate and firing (Gandolfi et al., 2024) and activity based (Rubin et al., 2011) models which have been proven capable of integrating different functional of the respiratory system [see (Molkov et al., 2017) for a complete list of models]. Among these, the activity-based models are the most widely employed to encompass the features of respiratory nuclei that can be abstracted as coupled oscillators. However, despite a series of advantages like the reduced computational load, the speed in the implementation and in general a reliable description of static oscillations, these models have been criticized for the reduced number of parameters that makes the system hardly modulated and consequently it is poorly adaptable to temporal dynamics.

In clinical practice, the artificial control of respiration is fundamental when arterial oxygenation and/or the elimination of carbon dioxide fails. In these cases, the mechanical control of ventilation coupled with ECMO is strongly recommended (Zhang et al., 2017). Nevertheless, the recovery from ECMO and the restoration of physiological ventilation, namely the respiratory weaning, is particularly challenging, since there is still controversy on the degree of mechanical ventilation support and the rate of transition towards the sole native lung activity (Pham et al., 2023). On one side the persistence of mechanical support may improve the lung recovery, on the other side lung recruitment may accelerate lung recovery. A unique procedure to obtain the optimal respiratory weaning cannot be established and each patient has its own algorithm that combines different parameters. In all cases in fact, clinicians and healthcare teams must assess respiratory, blood and cardiac functions before removing ECMO and restoring lung physiology. Among the parameters that need to be monitored during weaning the flow rate and blood gases are the most important and there are no particular settings for mechanical ventilation that are recommended. It is a widespread opinion that the control of the lung activity based on the natural control of a patient’s physiological parameters may help personalize the algorithm to adopt during weaning (Pham et al., 2023; Chen et al., 2022). It has been recently shown in preclinical evaluations that a physiological control of breathing to bypass spinal cord injuries (Siu et al., 2020) or to restore normocapnia after anesthesia (Zbrzeski et al., 2016) can be achieved through the implementation of bio-inspired artificial neural networks. Notably, similar applications have been developed to exploit the capability of spiking neural networks (SNNs) in reproducing motor CPGs. In these cases, SNNs are used, for instance, to drive robotic actuators for automatic motor control by adopting low power high-efficiency neuromorphic systems (Donati et al., 2014; Gutierrez-Galan et al., 2020) in invertebrates suggesting the feasibility of the present idea.

### Spiking neural model to capture the complexity of the respiratory circuit

After several years since the first development of a neuron model (Dayan and Abbott, 2001), there is increasing consensus that spiking networks can be used not only to capture the physiological features of biological circuits but also to implement cybernetics solutions for real-world applications. In particular, given the importance of keeping physiological constraints, the use of spiking neural networks in the field of medicine is crucial. Beside the reduced computational load, SNNs allow in fact to adapt dynamically to specific patient’s parameters by acting on single neurons and synapses. Moreover, the embedding of synaptic plasticity rules within the network enable the automatic tuning of the system according to the current working regime. We have witnessed in the last decade an increasing number of applications, ranging from digital brain twin (Gandolfi et al., 2023) to neuroprosthetic (Raspopovic et al., 2014) and neurorehabilitation (Romeni et al., 2025).

Given these premises, we are firmly convinced that spiking neuron models represent a promising tool for capturing the complex architecture of the respiratory system, without compromising the physiological fidelity of the main neuronal classes involved. Compared to rate-based models, SNN preserve the temporal resolution needed to investigate the synchronization mechanism. At the same time, extended Integrate-and-fire models significantly reduce the computational cost compared to Hodgkin Huxley models, while still reproducing a wide range of electrophysiological properties. For instance, spiking models can closely reproduce key features of excitatory neurons of the preBötC (Butera et al., 1999), including autorhythmicity (Jasinski et al., 2013), frequency adaptation – critical for generating a rhythm in purely excitatory networks- and, depending on the theory, autonomous bursting. These properties make them particularly well-suited for simulating the neural basis of rhythmogenesis of breathing and most importantly can be adopted as integrated solutions for clinical applications exploiting their full potentialities as biomedical tools. Interestingly SNNs can be embedded with synaptic plasticity learning rules to exploit adaptable behaviors required by dynamic systems evolving in time (Mapelli et al., 2022). There is growing evidence that SNNs can be efficiently employed to reproduce respiratory CPGs since similar approaches allowed to reproduce motor CPGs (Yamazaki et al., 2022).

**Figure 2:**
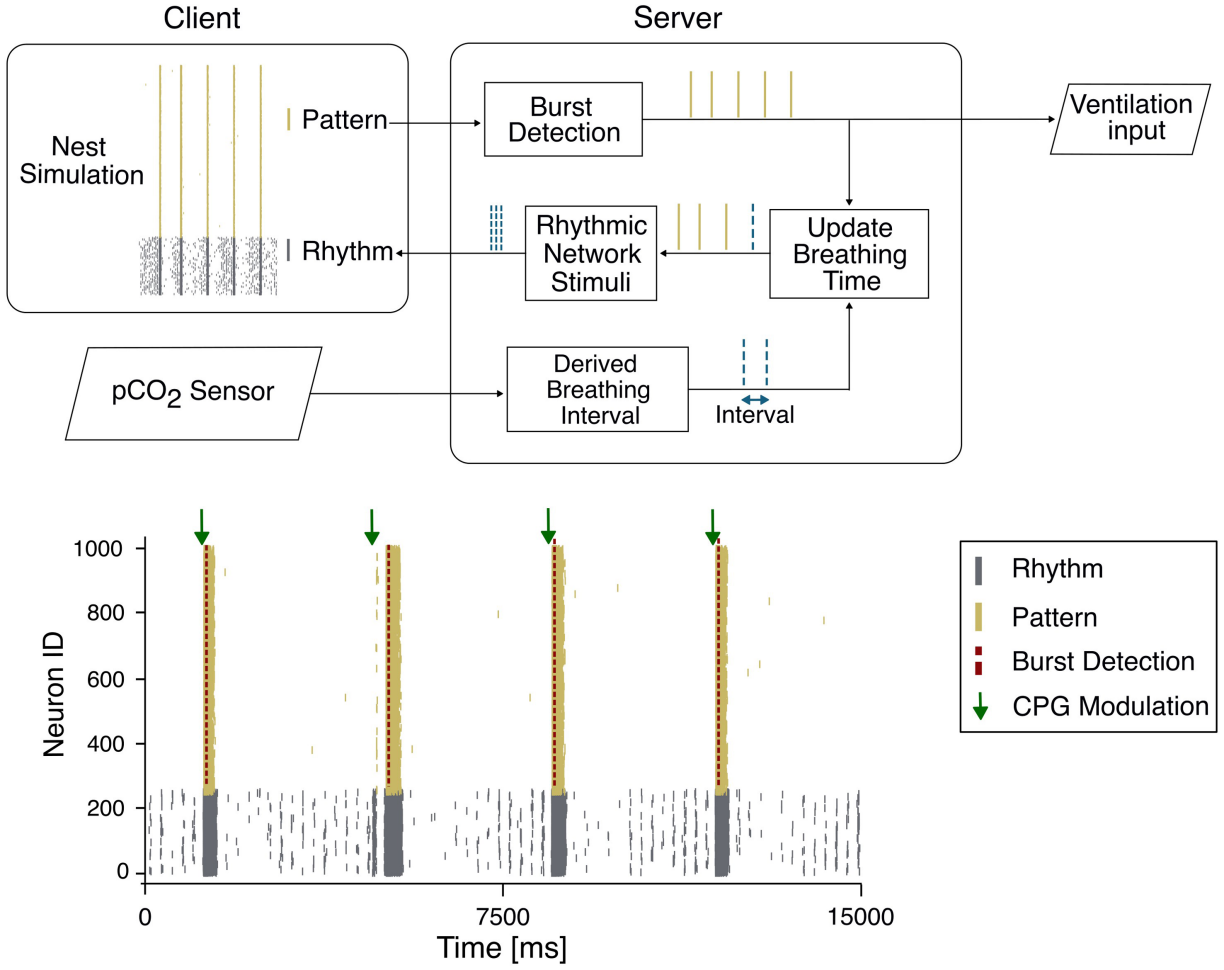
**(Top)** Closed-loop system relying on the synchronization between a client, responsible for the simulation and a server collecting the burst detection and the data from the sensor. The rhythmogenesis neurons are represented as two populations: pattern (yellow) and rhythm (gray). The pattern population generates the output bursts while the rhythm population receives the modulation input from the server. **(Bottom)** Closed-loop system dynamics, where the rhythmic network (gray) is driven by a pCO2 modulated input (green arrow) based on the last detected burst (red dashed line) of the pattern population (yellow).

In the context of respiratory rhythmogenesis, a single spiking neuron model could be used to create the network responsible for the respiratory rhythm. The network could be progressively expanded to further incorporate additional neuronal populations involved in the modulation of breathing. For instance, despite it has been shown that inhibitory interneurons are not necessary to instantiate breathing rhythmogenesis, their inclusion within the rCPG could reduce rhythm variability and therefore enhance regularity. In a wider perspective, the rCPG should include not only neurons responsible for the rhythmogenesis but also neuronal populations involved in the chemoreception like the RTN, which modulate respiratory activity in response to changes in CO_2_ levels, or NTS modulating their firing according to the levels of blood gases and pH. To substantiate our long-term view, we have recently attempted to investigate the rhythm generation and modulation of breathing, by implementing a spiking neural network using the NEST Simulator (Diesmann and Gewaltig, 2007) to reproduce rhythmogenesis. According to the most credited theories about respiratory rhythmogenesis, we have divided this purely excitatory network into two subpopulations: the “Rhythm,” which exhibits autorhythmicity, and the “Pattern,” which remains silent unless recruited by the Rhythm neurons (Figure 2 bottom panel). Beside the respiratory rhythmogenesis, which is consistent with a human eupneic breathing rate, to further support our vision the network has been integrated with a group of neurons corresponding to the chemoreceptive RTN, which increase the firing rate in response to elevated levels of partial pressure of blood carbon dioxide (pCO_2_) and synchronize the activity of the whole CPG network.

### Integrating spiking neural models into mechanical ventilation systems

We argue that biologically grounded spiking models, capable of processing biological stimuli while preserving core neural functions, represent the next step in guiding mechanical ventilation for patients lacking, or temporarily deprived of, autonomous respiratory function. This research direction reflects the growing interest in applying computational modelling to clinical technologies, as recognized by the 2024 priority of the U.S. FDA Centre for Devices and Radiological Health, which encourages the development of simulations to inform the decision-making process. Closed-loop strategies that respond dynamically to patient-specific signals are already emerging, particularly in systems that guide mechanical ventilation via real-time diaphragmatic stimulation either in the form of neuromorphic systems directly implanted within patient nervous systems (Dupeyroux et al., 2021) or in the form of artificial neural networks to externally control respiration (Siu et al., 2020). Building on this trajectory, we believe that in the next few years SNNs can play a pivotal role in the context of integrated respiratory support strategies - particularly in systems combining extracorporeal membrane oxygenation (ECMO) with mechanical ventilation. In such scenarios in fact, where artificial and native lungs work in parallel, the ability of a central pattern generator (CPG) to drive ventilation according to continuous physiological inputs could mark a paradigm shift and improve respiratory weaning. This integration may not only optimize ventilatory support during the acute phases of mechanical ventilation but also enable more adaptive, patient-specific interventions that better preserve and restore endogenous respiratory functions during the recovery phases. In the complex framework of artificial control of the. Respiratory system, the implementation of integrated hardware solutions is becoming crucial to assist clinicians in the critical care unit and in surgical practice. Integrated mixed analog/digital circuits to support spiking neural networks have been proposed for a several applications and more recently hardware neuromorphic platform have specifically developed to mimic the mammalian coupling of heart with breathing rate (Krause et al., 2021), the respiratory motoneuron functions (Abu-Hassan et al., 2019) or a complete system integrating the three-phase respiratory network and the heart champers rhythm (Donati et al., 2021). These solutions embed the concept of bioelectronic medicine which is increasingly becoming an important tool that bidirectionally integrates nervous signals with in-silico circuits that mimic biological neuronal networks. This approach soon could be adopted to both repair diseased circuits and to emulate neural functions with biomedical implants with a very high energy efficiency and low-power demands.

### Closed-loop respiratory rate control system

In the wide framework of real-time applications, the main requirement is that the model can be interfaced with external inputs not strictly generated within the simulation environment itself. This need has already emerged in other neural modelling studies, where the model had to interact with signals originating from other brain regions (Gambosi et al., 2024). In the perspective of a real time modulation of the respiratory rate, we envisage the combination of the respiratory CPG model with an external input corresponding to a physiological parameter like, for instance, the levels of pCO_2_ (Cattini et al., 2021) or pH (Cattini et al., 2020) which reflect the patient’s metabolic demand and drive the respiratory modulation (Figure 2 top panel). The pCO_2_ values, detected by using optical sensors based on fluorescence (Cattini et al., 2021) or near-infrared spectroscopy (Guglielmini et al., 2025; Hakimi et al., 2022), could be used to tune the SNN activity according to specific equations (Duffin et al., 2000) that convert values detected by sensors into trains of digital action potentials. As a results, the synchronization of the CPG-SNN that could be recognized by burst detection algorithms running in continuous and consecutive time windows, would be extracted as a digital output in turn controlling mechanical ventilation. The possibility to introduce computer-aided control of ventilation system only recently has become object of debate (Hariharan et al., 2025). In our vision, the output generated by the CPG model can be combined with the pCO_2_ values detected from external sensors and collected by a server, which must be synchronized with the client simulation. At every simulation step, the client and the server exchange data (Figure 2 top panel). A control feedback, based on the registered network state and the sensor input, can close the control loop, to ultimately modulate the CPG based ventilation with soft real time constraint. In this control loop, the actual real time is determined by the discrepancy between the simulation resolution, the computational time and the sensor sampling frequency. A putative closed-loop simulation test is shown in Figure 2, where the CPG network delivers, in the form of burst activity, the input to the ventilation system while receiving from a pCO₂ sensors a modulatory input that is converted into spike sequences before being conveyed back through RTN. The communication in real time can be easily achieved through software and hardware optimization processes. In the present example, a simulation with 1,000 e-GLIF neurons running on a conventional CPU, is optimized through parallelized multi-process computation to simulate 1 min of CPG activity in less than 45 s, therefore demonstrating the capability to instantiate real-time closed loop. During the monitoring of a patient, no delay between effective time and simulation time would be accumulated since the adopted communication protocol can simulate temporal blocks updating digital inputs and outputs in a quasi-continuous manner. Moreover, in the I/O communication procedure, potential sources of delay could be due (i) to burst detection and (ii) to the conversion of measured pCO_2_ values into frequency stimulation. In both cases the computation is performed in less than 30 microseconds [(i) 28 10^−6^ s; (ii) 28 10^−6^ s] leaving enough temporal resource for a dynamic regulation of respiratory activity by artificial CPG occurring on the scale of seconds. The transition from state to state would be solely dependent on the sampling rate of the pCO_2_ while the change in respiration rate would occur from breath to breath. Furthermore, we can envisage that the scaling up of the SNN by incorporating additional neuronal populations like BötC or NTS would require improvements in the simulation environment because of the increased computational load. The use of accelerated hardware like GPUs (Vieth et al., 2024) or ultimately the implementation of neuromorphic solutions either in the form of commercially available devices like Loihi (Michaelis et al., 2022), Dynap-se (Richter et al., 2024) or Akida (Lutes et al., 2025) would certainly allow to easily sustain the computational costs. Alternatively, dedicated solutions based on dedicated neuromorphic architectures (Florini et al., 2024; Benatti et al., 2023) shall further accelerate the time for computation and simulation to implement real-time, closed loop respiratory control (Siu et al., 2020).

## Conclusion

In the context of intelligent weaning, the management of an effective transition from mechanical ventilation and ECMO to physiological breathing is crucial to avoid abrupt transition. In this context, the possibility to predict patient’s respiratory capacity, could implement an intelligent artificial solution to be adapted on patient’s needs. To this aim, biologically grounded spiking neural network models could open new directions for the development of closed-loop respiratory support systems that could assist clinicians and healthcare teams during respiratory weaning. The inputs from spiking networks could provide valuable parameters to personalize the patient’s algorithm for ECMO recovery. We therefore envisage that spiking models capable of generating respiratory pattern that closely mirrors natural behaviors could provide physiological support in cases of impaired or absent autonomous breathing, such as during full or partial ECMO recovery. In the future, additional nuclei could be included in the artificial CPG to mimic the physiological centers that participate in the regulation of respiration. The expansion of the network, on one side would allow to directly modulate breathing according to metabolic parameters which are related to patient’s needs, on the other side would inherently increase the computational load and potentially limiting the chances to perform real time closed-loop control. However, optimization procedures and the introduction of neuromorphic hardware shall enable the implementation of large network configurations. The mimicking of homeostatic responses by the biological CPG that naturally operates in a negative feedback modality integrated into a closed-loop framework would enable the optimization of ventilation parameters based on real-time metabolic demand. We can envision that similar solutions based on biological controllers could be adopted in the routine of clinical practice to offer the opportunity of a massive minimization of risks associated with over- or under-ventilation by tailoring support to individual, real-time needs.

## Data Availability

The original contributions presented in the study are included in the article/supplementary material, further inquiries can be directed to the corresponding authors.
